# A novel home-based method for preparing suspensions of anti-TB drugs

**DOI:** 10.5588/ijtld.23.0165

**Published:** 2023-11-01

**Authors:** R. Taneja, M. C. Nahata, J. Scarim, P. G. Pande, A. Scarim, G. Hoddinott, C. L. Fourie, R. K. Jew, H. S. Schaaf, A. C. Hesseling, A. J. Garcia-Prats, K. Rao Inabathina

**Affiliations:** 1 Global Alliance for TB drug Development (TB Alliance), New York, NY; 2 Institute of Therapeutic Innovations and Outcomes, Colleges of Pharmacy and Medicine, The Ohio State University, Columbus, OH; 3 JSAS Services, Tucson, AZ; 4 Desmond Tutu TB Centre, Department of Paediatrics and Child Health, Faculty of Medicine and Health Sciences, Stellenbosch University, Tygerberg; 5 Metro TB Complex, Department of Health, Pretoria, South Africa; 6 Institute for Safe Medication Practices, Plymouth Meeting, PA; 7 Department of Pediatrics, University of Wisconsin School of Medicine and Public Health, Madison, WI, USA

**Keywords:** dosing device, pediatric, age-appropriate dosing, dysphagia, XTEMP-R

## Abstract

**BACKGROUND:**

Tablets are the most widely available dosage form for the treatment of TB; however, adult tablets fail to meet the needs of young children who cannot swallow these tablets or require dose titration. We tested a new, simple device (XTEMP-R^®^) and the methodology for converting tablets of TB drugs into a homogeneous suspension for home use by children and caregivers.

**METHODS:**

XTEMP-R is a new device used for converting tablets into liquid preparations. Four TB drugs – pretomanid, delamanid, clofazimine and bedaquiline – were dispersed in the device utilizing water and simple syrup. The reproducibility of accurately delivering aliquots from the suspension upon preparation and upon redispersion after storing for 2 days was studied.

**RESULTS:**

Suspensions of each of the drugs tested were easily prepared in about 10 min and were visually uniform in consistency. Dosages in 2 and 5 mL were assessed in suspension, and those in 5 mL tested upon redispersion after 2 days. The observed range for these dosages spanned from 94.6% to 101.1% of the theoretical concentration for the suspensions under examination. The cleaned device had no detectable residual drug.

**CONCLUSION:**

XTEMP-R can be used at home by caregivers to prepare doses of suspensions accurately for children and patients who cannot swallow tablets.

## Abstract (fr)

**CONTEXTE:**

Les comprimés sont la forme posologique la plus répandue pour le traitement de la TB; cependant, les comprimés pour adultes ne répondent pas aux besoins des jeunes enfants qui ne peuvent pas les avaler ou qui ont besoin d’un ajustement de la dose. Nous avons testé un nouveau dispositif simple (XTEMP-R^®^) et la méthodologie pour convertir les comprimés de médicaments antituberculeux en une suspension homogène pour une utilisation à domicile par les enfants et les soignants.

**MÉTHODES:**

XTEMP-R est un nouveau dispositif utilisé pour convertir les comprimés en préparations liquides. Quatre médicaments antituberculeux – pretomanid, delamanid, clofazimine et bedaquiline – ont été dispersés dans le dispositif en utilisant de l’eau et du sirop simple. La reproductibilité de l’administration précise d’aliquotes de la suspension lors de la préparation et de la redispersion après un stockage de 2 jours a été étudiée.

**RÉSULTATS:**

Les suspensions de chacun des médicaments testés ont été facilement préparées en 10 min environ et leur consistance était visuellement uniforme. Les dosages de 2 et 5 ml ont été évalués en suspension, et ceux de 5 ml ont été testés lors de la redispersion après 2 jours. La fourchette observée pour ces dosages s’étendait de 94,6% à 101,1% de la concentration théorique pour les suspensions examinées. Le dispositif nettoyé ne présentait aucun résidu de médicament détectable.

**CONCLUSION:**

XTEMP-R peut être utilisé à domicile par les soignants pour préparer avec précision des doses de suspensions pour les enfants et les patients qui ne peuvent pas avaler de comprimés.

Rifampicin-resistant TB (RR-TB), including multidrug-resistant TB (MDR-TB), continues to be a serious public health threat globally, and is estimated to cause up to 32,000 incident cases in children aged <15 years each year.^1^ In 2018, the WHO updated its guidance on the management of RR/MDR-TB. Chief among these recommendations was the re-prioritizing of second-line drugs into Group A, B, and C medications. Although the data supporting this drug re-classification largely came from adults, the principles of regimen design also apply to children.^2^

There have been some positive recent developments in pediatric TB drug formulations and administration technologies for low-resource settings. Access is improving, but there are still children who cannot access age-appropriate pediatric formulations, especially in low- and middle-income countries (LMICs).^3^–^5^ Although for the youngest pediatric patients (0–5-year olds), caregivers in many LMIC settings typically prefer liquid formulations, e.g., suspensions or syrups, tablets and capsules remain the default standard for TB drug development.^6^ Manipulation of these tablets based on age or body weight is required for dosing children or for patients suffering from dysphagia,^7^ when dispersible tablets or other liquid formulations are unavailable. This involves the physical alteration of the dosage form by tablet splitting, crushing, or grinding using cutting, crushing or grinding devices, or mortar and pestle.^7^ However, these practices often result in drug loss, lack of content uniformity, and dosing inaccuracy, while aerosolization of the drug powder also poses a safety risk to the caregiver. Thus, there is a need for convenient and safer methods to prepare liquid formulations from tablets.^7^–^10^

In a series of recently published papers, we have described the process for preparing extemporaneous formulations of key second-line TB drugs – bedaquiline (BDQ), clofazimine (CFZ), delamanid (DLM), and pretomanid (Pa) – in a pharmacy or a dispensary setting under the supervision of a pharmacist.^11^–^14^ In this paper, we describe a novel simple device and the methodology of using this device to convert BDQ, CFZ, DLM, and Pa tablets into liquid suspensions at home. This is especially beneficial for patients who do not have access to child-friendly or dysphagia-friendly formulations, or easy access to compounding pharmacies.

We developed a novel, simple device (XTEMP-R^®^; (Ami Polymers, Mumbai, India) for converting tablets into a homogenous liquid suspension without crushing, grinding, or splitting the tablets; multiple medications could potentially be combined. Flavors and sweeteners may be added to the liquid suspension to enhance palatability. While the methodology described in this paper pertains to the selected TB drugs, it could also be applied to medications used in other therapeutic areas.

This device and methodology are designed for immediate-release tablets. It is not suited for modified-release, delayed-release, or controlled-release tablets and capsules. In this paper, the term ‘tablet’ refers to immediate-release tablets. The term ‘dispersion’ refers to the disintegration of tablets and dispersing them in the liquid media, resulting in a suspension. The terms ‘dispersion’ and ‘suspension’ convey the same meaning.

Here, we describe methods for utilizing the XTEMP-R device to prepare suspensions of key second-line TB drugs from available adult tablet formulations, and the uniformity and dose reproducibility of the resulting suspensions.

## METHODS

### XTEMP-R^®^ device

**Figure 1:**
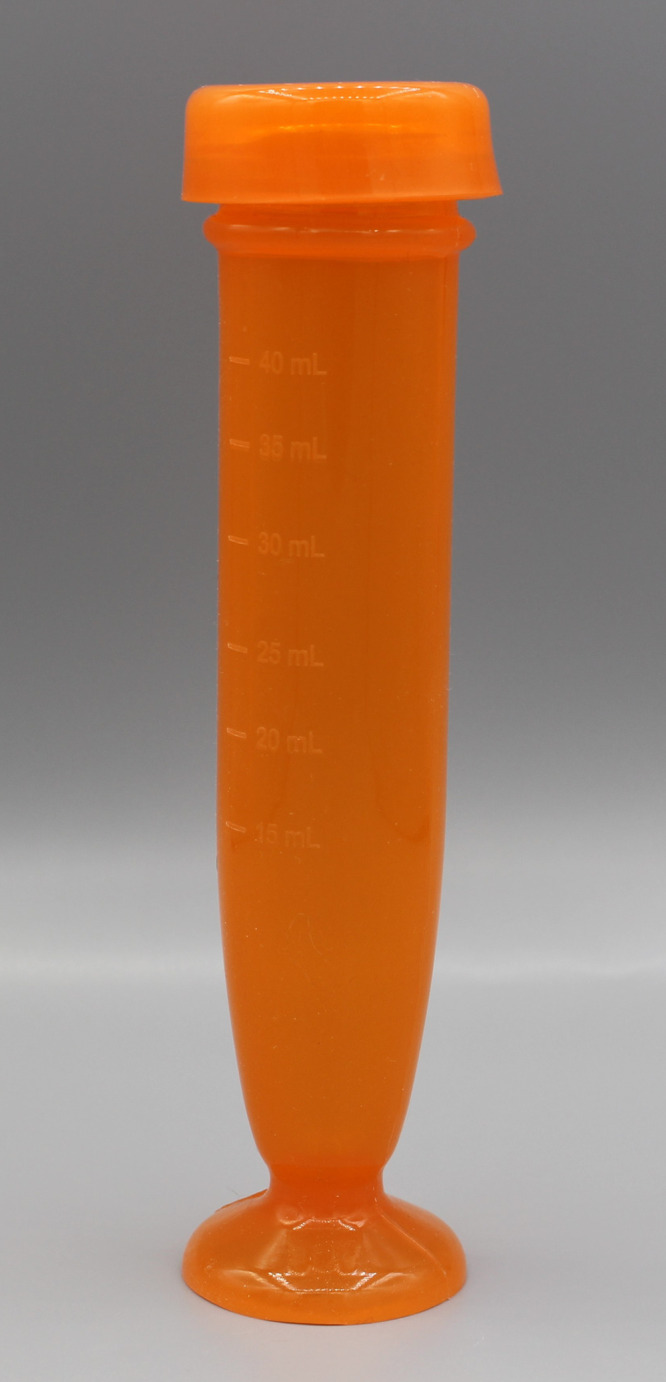
XTEMP-R^®^ device.

We designed and developed the XTEMP-R device (described elsewhere).^15^ The device comprises a flexible receptacle or tube, a tight-fitting cap, and a suction cup base (Figure 1). The soft walls of the device are made of biocompatible, low-hardness, high-consistency silicone rubber (Wacker Chemie, Munich, Germany). The platinum-cured silicone rubber complies with the FDA 21 CFR 177.2600, the German Bfr XV, French *arrêté du 25 novembre 1992*, RoHS (Restriction of Hazardous Substances), TSE/BSE (bovine spongiform encephalopathy/transmissible spongiform encephalopathy) free, USP Class VI, and ISO 10993 certifications. Based on these certifications, the material of XTEMP-R device is safe for repeated use with food. The devices can be manufactured in a class 10,000 clean room using a compression molding process (Ami Polymers). The XTEMP-R device has an internal diameter of 22 mm and is 140 mm in height. The device has volume markings on the outside at 5-mL intervals, starting at 15 mL up to the maximum capacity of 40 mL. Extreme temperatures have minimal effect on the physical properties of the device, as its service temperature ranges from -60°C to 230°C and is dishwasher-safe. The XTEMP-R device is easy to squeeze between the fingers for manual dispersion of the tablets and is leak-resistant.

### TB drugs and concentrations

Four TB drugs that are commonly prescribed for TB treatment were tested for ease of preparation at home. One drug was selected from each of the WHO second-line agents in group A (BDQ), B (CFZ) and C (DLM), as well as Pa, which has recently been included by the WHO as part of a treatment regimen for adults. We follow previously described preparation procedures for compounded formulations of these drugs in simple syrup.^11^–^14^ The concentrations selected for the four drugs were BDQ 20 mg/mL, CFZ 10 mg/mL, DLM 5 mg/mL, and Pa 20 mg/mL, based on discussions with clinicians and pharmacists in the field and the age-appropriate doses provided in the Field Guide by the Sentinel Project.^2^ Within these concentrations, it is possible to accurately measure dosing volumes at home using oral syringes, spanning from half a teaspoon (2.5 mL) to 4 teaspoons (20 mL).

### Preparation of suspensions

Commercially available adult-formulation tablets of the four TB drugs were used to prepare separate suspensions of each drug. Twenty mL of each suspension was prepared using the following number of tablets per drug: 4 BDQ 100 mg tablets, 2 CFZ 100 mg tablets, 2 DLM 50 mg tablets, and 2 Pa 200 mg tablets.

**Figure 2:**
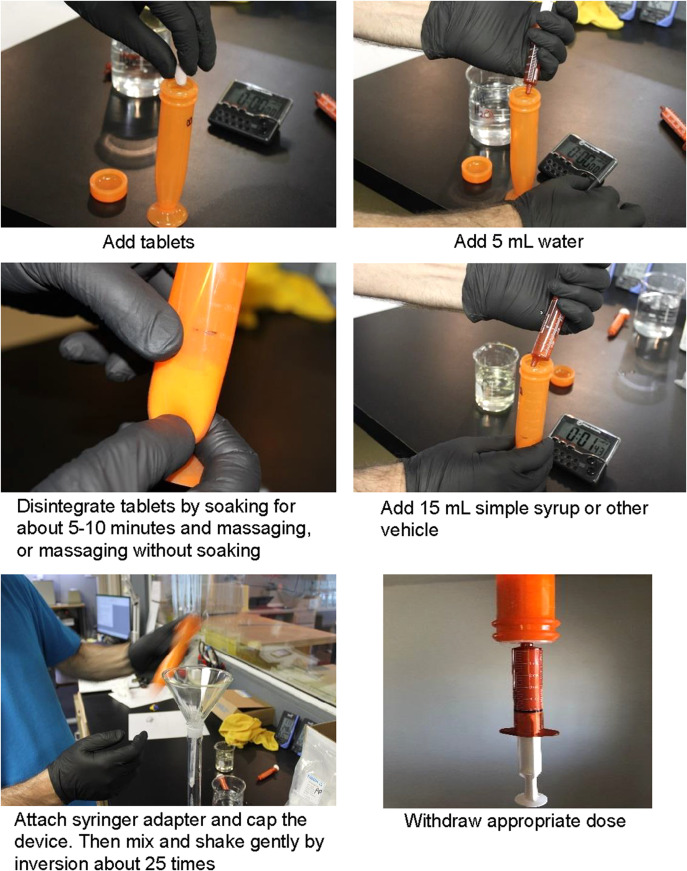
Instruction sheet for the preparation of the suspensions at home.

The required number of tablets were soaked in 5 mL of water for about 5–10 min in the XTEMP-R device to allow the tablets to disintegrate. The tablets were then massaged in the XTEMP-R device externally by rubbing the soft walls at the bottom of the device between the fingers to mix the disintegrated tablets mass until there was no grittiness and it was smooth to the feel. The massaging can take on average from a few seconds to less than a minute, depending on the tablet. This was followed by the addition of 15 mL of simple syrup to the device. A syringe adapter was then attached to the mouth of the device and the device was capped. The suspension was shaken gently by inversion about 25 times. This resulted in 20 mL of suspension of each drug at the concentrations specified above (See Figure 2 for preparation instructions).

### Visual observation

The prepared suspensions were shaken, and an aliquot of each was poured onto a glass petri dish. The suspensions were visually examined for uniformity and presence of large particles, agglomerates, or clumps.

### Dose reproducibility and redispersibility in XTEMP-R

A total of three suspensions per drug were prepared as described. For dose reproducibility, 2 mL and 5 mL doses were withdrawn from each of the three suspensions of each drug. For re-dispersibility, the suspensions were stored at ambient conditions for 2 days. The suspensions were then shaken by inversion about 25 times, and a dose of 5 mL withdrawn from each of the three suspensions of each drug.

### Analytical methods

Stability-indicating high-performance liquid chromatographic (HPLC) methods were developed for each drug and verified using forced degradation studies, as previously described.^11^–^14^ Details of the analytical methods are given in Table 1 (Supplementary Data).

**Table 1:** Stability-indicating HPLC methods for bedaquiline, clofazimine, delamanid and pretomanid

Parameter	Bedaquiline	Clofazimine	Delamanid	Pretomanid
Instrument	Hitachi L-2100 pump (Hitachi; Tokyo, Japan), Shimadzu (Shimadzu Corp; Kyoto, Japan) SPD-10AVP detector, Shimadzu SCL-10AVP Controller (Shimadzu Corp), Shimadzu CTO-AVP Column Heater (Shimadzu Corp), SRI Instruments PeakSimple chromatography data system (SRI Instruments; Torrance, CA, USA)			
Column	Waters Sunfire^®^ (Waters; Dresden, Germany) (C18, 4.6 mm × 100 mm, 3.5 μm)			
Wavelength	225 nm	283 nm	223 nm	234 nm
Column temperature, °C	40°C			30°C
Flow rate, mL/min	1.5			1.0
Injection volume, µL	10			20
Syringe filter	Polypropylene, 0.45 µm			
Stock standard concentration	0.5 mg/mL	0.5 mg/mL	0.25 mg/mL	0.5 mg/mL
Stock preparation diluent	Water: acetonitrile (50:50)	Acetic acid: water: methanol (75:425:500)	Water: acetonitrile (50:50)	Water: acetonitrile (50:50)
Working standard concentration, mg/mL	0.02	0.02	0.025	0.025
Working preparation diluent	Water: acetonitrile (50:50)	Water: acetonitrile (50:50)	Water: acetonitrile (50:50)	Water: acetonitrile (50:50)
Mobile phase	Water: acetonitrile: TFA (500: 500:1)	Water: acetonitrile: TFA (550:450:1)	Water: acetonitrile: TFA (650:350:1)	Water: acetonitrile: TFA (550:450:1)
Run time, min	6	8	10	9
Peak, min	4.5–5.5	6–7	8	7
Integration	Peak area method using PeakSimple data acquisition system, 5 Hz		

HPLC = high-performance liquid chromatographic; TFA = trifluoroacetic acid.

### XTEMP-R device cleaning

After use, the XTEMP-R devices were cleaned using water and a mild liquid dishwashing detergent. To evaluate whether residue drug might remain after cleaning, a placebo was prepared by mixing 5 mL of water with 15 mL of simple syrup in the devices after cleaning, and tested for the presence of the drug prepared in each device.

### Outcome measures

The ease of preparation of the suspensions was determined by the time required for completing the preparation. The consistency of the suspensions was assessed based on the visual appearance of the suspensions. Dose reproducibility was determined by testing the aliquots from the prepared suspensions and re-dispersibility upon shaking after 2 days. The doses in aliquots withdrawn were reproducible, and mean potency was deemed acceptable if the measured drug concentrations of each of the three aliquots tested were within 10% of the theoretical value^16^ at each time point. The cleaning procedure was considered acceptable if no residual drug was detected in placebo preparations made in the cleaned devices.

## RESULTS

Each suspension of the four drugs tested – BDQ, CFZ, DLM, and Pa – were easily prepared in about 10 min. The dispersions of all four drugs tested in the petri dish were visually uniform and no clumps or lumps were present. Results for dose reproducibility from the 5 mL and 2 mL aliquots upon suspension, and from the 5 mL aliquots upon redispersion are shown in Table 2. Assay results of all individual BDQ aliquots ranged from 95.2% to 98.7% of the theoretical concentration, with means ranging from 95.6% to 98.2%. Assay results of all individual CFZ aliquots ranged from 94.8% to 98.5% of the theoretical concentration, with means ranging from 95.4% to 97.6%. For DLM, the assay results of individual aliquots ranged from 98.9% to 101.1% of the theoretical concentration, with means ranging from 99.4% to 100.5%. For Pa, assay results of individual aliquots ranged from 94.6% to 99.0% of the theoretical concentration, with means ranging from 95.2% to 98.4%.

**Table 2:** Dose reproducibility of aliquots withdrawn from suspensions

Suspension	Proportion of theoretical dose dispensed			
	Day	0		2
	Aliquot	5 mL	2 mL	5 mL
		%	%	%
BDQ, 20 mg/mL	1	95.8	98.6	98.7
	2	95.7	98.4	97.1
	3	95.2	97.7	97.7
	Mean ± SD	95.6 ± 0.3	98.2 ± 0.4	97.8 ± 0.7
CFZ, 10 mg/mL	1	94.8	97.2	96.4
	2	96.3	97.2	95.4
	3	95.2	98.5	95.3
	Mean ± SD	95.4 ± 0.6	97.6 ± 0.6	95.7 ± 0.5
DLM, 5 mg/mL	1	99.8	101.1	100.3
	2	99.5	100.7	100.4
	3	98.9	99.8	100.8
	Mean ± SD	99.4 ± 0.4	100.5 ± 0.5	100.5 ± 0.2
Pa, 20 mg/mL	1	97.4	97.3	95.4
	2	96.2	98.8	95.7
	3	94.7	99	94.6
	Mean ± SD	96.1 ± 1.1	98.4 ± 0.8	95.2 ± 0.5

BDQ = bedaquiline; SD = standard deviation; CFZ = clofazimine; DLM = delamanid; Pa = pretomanid.

No residual drug was detected in samples of the placebo tested from the cleaned devices used to prepare all four drugs, although the devices that were used to prepare CFZ suspensions were stained due to the red color of the drug.

## DISCUSSION

Lack of access to appropriate formulations remains a challenge for dose administration of TB drugs in children and in patients with dysphagia, as well as patients in intensive care units. Tablet splitting and crushing are routinely employed in the field. However, there are challenges associated with these practices such as dosing inaccuracy, drug loss, lack of content uniformity, and hazard from aerosolized drug particles.^7^–^10^ Palatability is another challenge for administration and adherence. We recently described the methodology for the preparation of extemporaneous formulations of BDQ, CFZ, DLM, and Pa in a pharmacy/dispensary setting.^11^–^14^ However, access to a pharmacy may be limited in many situations. We have therefore developed a device and methodology that permits extemporaneous formulation preparation at home by the caregiver or patient.

XTEMP-R is a simple mechanical device designed to facilitate and expedite the disintegration and dispersion of tablets in a small volume (<10 mL) of water. This device is constructed with low hardness silicone, making it pliable yet durable. It can be gently squeezed with the fingers to facilitate the dispersion of tablets. The squeezing and massaging actions facilitate the interaction of water and the super disintegrants present in the tablet, thus promoting its dispersion. Super disintegrants are routinely included in tablets to aid in the break-up of the compacted mass upon ingestion and contact with a liquid in the digestive tract.

The suspensions were prepared easily in about 10 min, with about 5–10 min of soaking time. To hasten the process of dispersion, the tablets and liquid in the tube can be massaged until the tablets are dispersed completely and contents are smooth to the feel. Once the tablets are dispersed, thickeners, sweeteners, and flavors can be added according to the patient’s preference, if necessary.

Simple syrup was selected as the vehicle in this study, as it can be easily prepared at home or obtained commercially. Syrup has a viscous consistency that prevents sedimentation, resulting in better uniformity of the dose dispensed, while the sweetness improves palatability. Simple syrup also has inherent preservative properties and can be prepared in bulk and stored for use over several days to mix with drugs at the time of dosing.

A simple and reproducible procedure using XTEMP-R was developed to convert tablets into a suspension. The disintegration of the tablets and the preparation of the suspension were safely performed in a contained environment within the same device. This method facilitated precise measurement and extraction of the necessary dosage using an oral syringe, or alternatively, the entire dose could be directly consumed from the same container. This offered a convenient dose administration option for pediatric patients, as well as patients suffering from dysphagia. The resulting dispersion also permitted administration through feeding tubes.

As demonstrated by the results, adult-formulation tablets of the four TB drugs studied – Pa, CFZ, DLM and BDQ – could be dispersed homogeneously in water and simple syrup within 10 min. Accurate aliquots could be withdrawn reproducibly from the device immediately after the preparation and up to 2 days later at room temperature. This closely replicates the scenario that would take place in the patient’s home. It was not our intention to prepare suspensions for long-term storage.

Improved age-appropriate dose accuracy, ease of preparation, and use over 2 days are expected to result in improved adherence, better acceptability, and better treatment outcomes. The device is reusable, as it can easily be cleaned manually using liquid dishwashing detergent and water. The device is also dishwasher-safe. For colored drugs, such as CFZ, which stain materials with which they come in contact, the device may be dedicated for use for that specific drug. TB treatment requires multidrug treatment. This device could be used for the preparation and coadministration of multiple drug suspensions. However, compatibility data and further studies are needed to ensure that the appropriate dose of each drug can be administered in the volume that can be accommodated by the device.

### Limitations

This methodology is only suitable for immediate-release tablets containing super disintegrants. In addition, stability data on the drug and the suspending agent must be available. As certain drugs may stain the device, it is recommended that a dedicated device be used for preparing such suspensions. The device was tested in a laboratory setting simulating the process that would be used at home on the assumption that it would give similar results when prepared at home by caregivers.

## CONCLUSIONS

We have demonstrated that homogeneous suspensions could be prepared conveniently, safely, and expeditiously in a contained environment using the XTEMP-R device without any significant drug loss. This device offers a patient-friendly alternative for dosing patients who cannot ingest immediate-release tablets, including children, the elderly and intubated patients. This novel XTEMP-R device can also be utilized for aliquoting partial doses of Pa, CFZ, DLM or BDQ. Additional work is required to establish the utility of the device for the preparation of suspensions of other TB drugs and for combinations of TB drugs as multiple tablets prepared as one suspension. This work addresses a huge unmet need for appropriate dose administration in pediatric and dysphagia patients.
