# Supplementary material for: A novel home-based method for preparing suspensions of anti-TB drugs
Source: Int J Tuberc Lung Dis. 2023 Nov 1;27(11):810–5. doi: 10.5588/ijtld.23.0165 (PMC10599413; doi:10.5588/ijtld.23.0165)
Supplement: Supplementary file 1 [file iutld_ijtld_23.0165_supplementarydata1.pdf]

http://dx.doi.org/10.5588/ijtld.23.0165

SUPPLEMENTARY DATA

A novel home-based method for preparing suspensions of antituberculosis drugs

List of ingredients, supplies, and reagents

Materials

Bedaquiline Tablets: 100 mg, Janssen, TMC207, lot JGTSD00

Clofazimine Tablets: 100 mg, Macleods, batch NCG2107B

Delamanid Tablets: 50 mg, Deltyba, Otsuka, batch B1621135

Pretomanid Tablets (PA-824): 200 mg, Dr. Reddy's Laboratories, lot ET15057

Bedaquiline Fumarate Bulk Drug, WuXi AppTec, batch EQ18408-1-P1

Clofazimine Bulk Drug (GATB005), Sigma-Aldrich, item C885, lot 095K1864

Delamanid Bulk Drug (OPC-67683, GATB023), Cambridge Major Laboratories, lot 1294-31-3

Pretomanid Bulk Drug (PA-824), Dr. Reddy's Laboratories, lot AFBH000496

Distilled Water

Simple Syrup (or cane sugar and water to prepare simple syrup)

Cane Sugar, C&H, lot 70424C105, or equivalent

Reagents and Supplies

XTEMP-R® device

Syringe adapter for bottles, 24mm, Clark Container (obtained through US Plastics, item # 081879)

Oral Dosing Syringe(s), 5 mL and 20 mL, Baxa, Exactamed, Baxter, or equivalent

HPLC Column, Waters, Sunfire™, C18, 4.6 x 100mm, 3.5um, PN 186002553

Syringe filters, 13mm, 0.45um, polypropylene, Tisch, PN SF14705, or equivalent

Acetonitrile, HPLC grade, Spectrum, item HP412, lot 20020323, or Concord, item 8002H-04, lot 200316, or equivalent

Acetic Acid, Sigma-Aldrich, ACS Reagent grade, item 695092-4L, lot STBJ9517

Methanol, HPLC grade, Spectrum, item HP702, lot 20120223, or equivalent

Water, HPLC grade, Spectrum, item HP902, lot 20040697, or equivalent

Trifluoroacetic acid (TFA), HPLC grade, Sigma-Aldrich, item 302031-10X1ML, lot MKCL3567, or equivalent.
